# Letter order is not coded by open bigrams

**DOI:** 10.1016/j.jml.2013.03.003

**Published:** 2013-08

**Authors:** Sachiko Kinoshita, Dennis Norris

**Affiliations:** a Department of Psychology and ARC Centre of Excellence for Cognition and its Disorders (CCD), Macquarie University, Sydney, Australia; b MRC Cognition and Brain Sciences Unit, Cambridge, United Kingdom

**Keywords:** Orthographic representation, Letter order, Open bigrams

## Abstract

Open bigram (OB) models (e.g., SERIOL: Whitney, 2001, 2008; Binary OB, Grainger & van Heuven, 2003; Overlap OB, Grainger et al., 2006; Local combination detector model, Dehaene et al., 2005) posit that letter order in a word is coded by a set of ordered letter pairs. We report three experiments using bigram primes in the same-different match task, investigating the effects of order reversal and the number of letters intervening between the letters in the target. Reversed bigrams (e.g., fo-OF, ob-ABOLISH) produced robust priming, in direct contradiction to the assumption that letter order is coded by the presence of ordered letter pairs. Also in contradiction to the core assumption of current open bigram models, non-contiguous bigrams spanning three letters in the target (e.g., bs-ABOLISH) showed robust priming effects, equivalent in size to contiguous bigrams (e.g., bo-ABOLISH). These results question the role of open bigrams in coding letter order.

## Highlights

• Open bigrams are ordered-letter pairs that code local order.
• We tested two core assumptions of open bigram models using bigram primes.
• Reversed bigrams and bigrams spanning three letters produced robust priming.
• The results provide no support for the role of open bigrams in coding letter order.

## Introduction

An issue currently receiving much attention in visual word recognition research is how letter order is coded in orthographic representations. In alphabetic orthography, the number of letters is severely limited, and hence the reader is confronted with a myriad of anagrams like CAT and ACT, TRAP and PART, and DYSLEXIA and DAILYSEX (the last example was taken from Snowden, Thompson, & Trosvianko, 2006). Based on the analysis of English words in the Celex corpus, Shillcock, Ellison, and Monaghan (2000) reported that for short English words, almost one third of words are anagrams (for 3-letter words, 33%, for 4-letter words, 34% and for 5-letter words, 20%). Anagrams can only be distinguished by the different order of letters, and hence any model of word recognition needs to be able to explain how letter order is coded so as to allow anagrams to be distinguished.

Many models of word recognition, like the interactive-activation model (McClelland & Rumelhart, 1981), models based on the interactive-activation model such as the Dual Route Cascaded (DRC) model (Coltheart, Rastle, Peery, Ziegler, & Langdon, 2001) and the multiple read out model (MROM, Grainger & Jacobs, 1996) – as well as the original Bayesian Reader model (Norris, 2006), use the “slot-coding” scheme. In this scheme, there are separate slots for each possible letter position within a word, and letter identities are associated with specific slots. For example, the word “CAT” would be represented as C_1_A_2_T_3_, with the letter C associated with the position 1 slot; letter A in position 2, and letter T in position 3. In contrast, the word “ACT” would be represented as A_1_C_2_T_3_. This means that the letters C (and A) in CAT and ACT are effectively different letters (C_1_ and A_2_ in CAT, and C_2_ and A_1_ in ACT). Although the slot-coding scheme allows anagrams to be distinguished, it is now widely recognized that the scheme is challenged by various phenomena demonstrating that readers are tolerant of distortions of canonical order of the letters in a word. Such demonstrations include the “Cambridge email”, transposed-letter priming effect and the relative position priming effects.

The Cambridge email (also referred to as the “jumbled text”), circulated in the internet around 2003, was a text in which the letter order in many of the words was distorted (“Aoccrding to a rscheerch at Cmabridge Uinervisty…”). The fact that people were able to read the message with relative ease demonstrated that readers were tolerant of quite substantial departures from the canonical order of the letters in a word (for a more formal demonstration, see Velan & Frost, 2007 and Rayner, White, Johnson, & Liversedge, 2006). The transposed-letter (hereafter TL) priming effect (e.g., Forster, Davis, Schocknecht, & Carter, 1987; Kinoshita & Norris, 2009; Perea & Lupker, 2003) refers to the finding that a prime generated by transposing two adjacent letters in a word (e.g., *ju**gd**e*) facilitates the recognition of the base word (*JUDGE*) almost as much as an identity prime, and more than a prime generated by replacing the corresponding letters with other letters not in the word (two-substituted-letter/2SL prime, e.g., *ju**np**e*). In both the TL prime and the 2SL prime the slots corresponding to the third and forth letters have the wrong letter identities. Slot-coding models therefore wrongly predict that TL primes and 2SL primes should facilitate the recognition of base word (*JUDGE*) equally. A related problem with the slot-coding scheme is that it cannot capture the similarity between letter strings differing in length that contain the same sequence of letters like *PRAY* and *SPRAY*. Ample evidence exists that primes generated from the baseword by deleting (subset prime, e.g., *aprt-APRICOT*) or adding letters (superset prime, e.g.*, journeal-JOURNAL*) produce robust priming effects (Grainger, Grainier, Farioli, van Assche & Grainger, 2006; Van Assche & Grainger, 2006) provided that the general order of letters is preserved – which is referred to as the relative position priming effect.

Accordingly, in visual word recognition research, much effort is currently directed at developing an alternative to the slot-coding scheme. In the approach that we favor (e.g., Norris & Kinoshita, 2012; Norris, Kinoshita, & van Casteren, 2010; see also Gomez, Ratcliff, & Perea, 2008), the key assumption is that, in the early stages of orthographic processing, uncertainty exists in the coding of letter position/order due to noisy perceptual sampling. The “noisy position” assumption finds support in the visual perception literature. The perception of spatial order of elements in a multi-element array (a sequence of colored circles or a string of random letters) is limited by crowding, and observers make many localization errors of neighboring elements (Popple & Levi, 2005). According to the noisy slot model (Norris et al., 2010), then, when the TL prime ‘jugde’ is presented briefly, it is ambiguous whether G is to the left or the right of D. Combined with the assumption that readers are optimal Bayesian recognizers trying to discover the optimal mapping between the noisy representation of input and lexical entries, to the extent that there is some possibility that D precedes G, the TL prime jugde will match JUDGE to some degree. As “JUDGE” is the closest word, the masked prime ‘jugde’ will facilitate the recognition of JUDGE. In the same way, people are able to read the Cambridge email even if they know that the word isn’t the exact match; the closest word to “Uinervisty” is still “University”. In the noisy channel model, Norris and Kinoshita (2012) extended the assumption of noisy perceptual sampling to the presence/absence of letter objects. In this model, the relative position priming effects are explained similarly in terms of the readers trying to discover the optimal mapping between a sequence of letters and a noisy representation of linearly ordered letter objects with missing and spuriously inserted letter objects.

In the SOLAR/Spatial Coding model (Davis, 1999, 2010), order is represented as an activation gradient over all of the letters in the input, where the first letter has the highest activation and each subsequent letter has a progressively lower level of activation.^1^ This “spatial gradient” representation forms the input to the word recognition system. TL priming and relative position priming effects are both explained in terms of the similarity in the activation pattern of the gradient representation of the prime and target.

In both the noisy channel model (and its precursor, the noisy slot model) and the SOLAR/Spatial Coding model, there is only one level of orthographic representation – letters.^2^ Words are presented as an ordered sequence of letters. A different approach that relies on an additional level of orthographic representation that codes the relative order of two letters in close proximity has been adopted by several groups of researchers (e.g., Dehaene, 2009; Dehaene, Cohen, Sigman, & Vinckier, 2005; Grainger, Granier, Farioli, van Assche, & van Heuven, 2006; Grainger & van Heuven, 2003; Grainger & Whitney, 2004; Whitney, 2001, 2008). The present paper focuses on evaluating these “open bigram” models.

### Open bigram models

Open bigrams (OB) are ordered letter pairs (bigrams) which can be contiguous or non-contiguous: For example, the word CAT contains the contiguous OBs CA, AT and the non-contiguous OB CT. The key claim of OB models is that a word is coded as an unordered set of OBs, for example, CAT is coded as {AT, CT, CA}. Grainger and Whitney (2004) suggested that “open bigrams provide a convenient computational mechanism for representing relative position of letters in a string” (p. 58), and that they provide a natural explanation for experimental data demonstrating TL priming and relative position priming effects. Specifically, priming is assumed to be a function of orthographic similarity between the prime and the target, which is indexed by the number of OBs shared by the letter strings. For example, if all OBs are represented, JUDGE contains the following 10 OBs: JU, JD, JG, JE, UD, UG, UE, DE, GE and DG. The TL prime *jugde* shares all of the OBs bar DG, i.e., it has 9 out of 10 matches. In contrast, the 2SL prime *junpe* shares with the target only three OBs JU, JE, and UE, i.e., has 3 out of 10 matches. Accordingly, the TL prime is more similar to the target than the 2SL prime, leading to a greater priming effect. It is also easy to explain relative position priming effects as superset and subset primes that preserve the relative order of letters (e.g., *aprt-APRICOT*; *journeal-JOURNAL*) share a number of OBs.

A distinctive feature of OB models is that they postulate two levels of orthographic representations. In the alternative models of letter order coding (the Spatial Coding model, Davis, 2010, the noisy channel model, Norris & Kinoshita, 2012; the Overlap model, Gomez et al., 2008), there is only one level of orthographic representation – letters. In contrast, in the OB models there are at least two distinct levels of orthographic information: OBs, and letters from which OBs are constructed. This begs the question of whether the extra level of representation is justified.

There are no data that indicate that reading specifically involves open bigrams. Proponents of the open bigram models have appealed to neurobiological data as providing unique support for open bigrams, but a closer inspection reveals this is not the case. For example, Whitney (2008; see also Dehaene, 2009) described an fMRI study by Binder, Medlar, Westbury, Liebenthal, and Buchanan (2006) as showing that an area of left middle fusiform gyrus (the area dubbed the “visual word form area”, Cohen & Dehaene, 2004) is “uniquely sensitive to bigram probabilities” (p. 175). In fact, Binder et al. specifically pointed out that their manipulation of mean positional bigram frequency was correlated with single letter, bigram, and trigram probabilities and that they “have not attempted to parcel out brain responses as a function of sequence fragment length” (p. 740).

In defense of open bigram representations, Grainger and colleagues (e.g., Grainger & Dufau, 2012; Grainger & van Heuven, 2003; Grainger & Ziegler, 2011) have repeatedly appealed to the notion of location invariance. In their Binary OB model, the “alphabetic array” codes for the presence of a letter at a given location relative to eye fixation along the horizontal meridian, that is, the alphabetic array contains location-specific letter detectors. Grainger and Ziegler (2011) point out that for the purpose of location-invariant word recognition, the location-specific representation needs to be mapped onto a location-independent code: As they put it, “identifying a unique orthographic code requires knowledge about where a given letter is in the word, not on the retina” (p. 2). In the Binary OB model, this transformation is achieved at the level of open bigrams which are assumed to be location-invariant. That is, in the Binary OB model, the open bigram representations are motivated by the need to transform location-specific (retinotopic) letter representations into location-invariant representations to allow letters to be recognized irrespective of spatial location. This assertion begs a question, however. As pointed out by Whitney and Cornelissen (2008), the Binary OB model “does not specify the underlying mechanisms of this conversion” (p. 149): it is simply asserted that retinotopic letter detectors are converted into a location-independent bigram code. Moreover, there is no *a priori* reason why the letter detectors should be retinotopic and the open bigram representations location-invariant. Consistent with this, in other open bigram models, the assumptions are different. In SERIOL, Whitney and Cornelissen (2008) state that both the letter representations and open bigrams are location-independent. In the LCD model (Dehaene et al., 2005) on the other hand, both the letter representations and bigram representations are retinotopic (albeit with positional noise). Thus, contrary to Grainger and Ziegler’s (2011) suggestion, what they call the “hard problem of orthographic processing” (p. 2) – that of transformation of location-specific retinotopic visual information into a location-invariant word-centered orthographic code – does not require open bigram representations.

In sum, there are no data that provide unique support for the open bigram models, nor is there a theoretical reason for positing OB representations. Open bigrams were originally proposed as a convenient computational solution to account for the TL priming and relative position priming effects. However, there are now two computational models (the Spatial Coding model, Davis, 2010, and the noisy channel model, Norris & Kinoshita, 2012) that provide detailed simulations of these data. Unlike these models, OB models need to postulate two levels of orthographic representations, making them less parsimonious. Given this, it would be reasonable to ask whether there is any evidence that OBs are actually used to code letter order.

Surprisingly, to date, no study has tested this question empirically. The variety of OB models with their differing parameter values complicates the testing of their predictions, however, there are two assumptions shared by all OB models. The first is that letter order is coded by the presence of ordered letter pairs. This is the central tenet of open bigrams, and is straightforward to test empirically. A bigram prime comprised of letters contained in the word should facilitate the recognition of the word *provided that the letters are in the right order*; bigram primes with the letters in the wrong order should not produce priming.

The second assumption shared by all current OB models is that the number of intervening letters spanning the constituent letters in an OB is limited to two: For example, in the word JUDGE, JE is not represented because it spans three letters (U, D and G). In Dehaene et al.’s (2005) LCD model, this assumption was motivated by the notion of a neuronal hierarchy based on the size of receptive field. According to Dehaene (2009), visual word recognition is subserved by a neuronal hierarchy along the ventral visual pathway whereby neurons at each stage learn to respond to a conjunction of neuronal activity from the immediately preceding level. At the lowest level, local contrasts are coded, then progressively larger units are coded through oriented bars, local contours, case-specific letter shapes, abstract letter identities, local bigrams, and finally short words and morphemes. Within this hierarchy, at each step, the receptive field of the neurons broadens by a factor of two or three. Dehaene (2009) thus argued that “As a result, the letters in bigram detectors can tolerate only a small shift of about two or three letter positions. Thanks to their limited receptive field, bigram neurons only fire if the first letter of a pair is less than two letters away from the second. For instance, a neuron coding for the pair AM can react to the words “ham”, and “atom” but not to “alarm” or “atrium” (p. 157).

In the Binary OB model, the assumption limiting the number of intervening letters to two was motivated by data. Schoonbaert and Grainger (2004) reported that priming produced by a subset prime was not affected by whether a letter that occurred more than once in the target was also repeated in the prime (e.g., *balnce-BALANCE* vs. *balace-BALANCE*). They noted that this did not fit the fact that fewer open bigrams were shared between the prime and the target when the prime contains the repeated letter as in *balace*, but the results can be accommodated if a limit is imposed on the number of intervening letters. They also noted that this modification was successful in accounting for other data observed with subset primes (Grainger et al., 2006) which the original unconstrained model could not account for. In SERIOL, the limit on the number of intervening letters follows from the assumption that the connection weights between an OB unit and the target word are a decreasing function of the distance between the constituent letters. Whitney (2008) set the parameter values of adjacent bigrams to 1.0, open bigrams spanning one intervening letter to .8, open bigrams spanning two intervening letters to .4, and open bigrams spanning two letters or more to 0, “because the constituent letters are too far apart in the base word to activate these open bigrams” (p. 176). Thus, while the stated motivations are different, all current OB models share the assumption that the number of intervening letters between the constituent letters in an OB is limited to two. That there is a limit to the number of intervening letters that can span an open bigram follows naturally from the fact that open bigrams code local context.

The present study provides an empirical test of these two assumptions. Experiment 1 uses two-letter words as targets to test the effect of reversal. With two-letter words (e.g., OF, MY), the OB models predict no priming effects from bigram primes with reversed order (e.g., fo-OF, ym-MY), that is, they predict no TL priming effect, because these primes share no OBs with the target. Experiment 2 uses 7-letter words to test the distance assumption: bigram primes in which the constituent letters span 3 letters (e.g., BS in ABOLISH) should produce no priming. Experiment 3 combines the reversal manipulation and the distance manipulation in 7-letter words to provide a replication.

### Masked priming in the same-different task

Most previous studies investigating the coding of letter order used the masked priming procedure developed by Forster and Davis (1984). In this procedure, a trial consists of a sequence of three events: (1) a forward mask consisting of # symbols (#####), (2) a prime presented in lowercase letters presented briefly (usually 40–60 ms), and (3) a target, to which a response is required, presented in uppercase letters. The forward mask, prime and target are presented in the same location, hence the prime is forward masked, and backward-masked by the target, so that it is not consciously recognized. It is widely assumed that this feature of masked priming procedure makes it well-suited to studying the automatic aspects of orthographic processing, free of strategic use of primes.

In testing masked priming here, we chose the cross-case sequential same-different task, rather than the lexical decision task typically used in previous studies. In this task, a referent (in lowercase letters) is presented in advance of the target, and the participant’s task is to decide whether the target (presented in uppercase letters) is the same as, or different from, the target. Because the referent and the target are presented in different case, the decision cannot be based on physical identity. Norris and Kinoshita (2008; Kinoshita & Norris, 2009; Norris et al., 2010) adopted the Forster and Davis masked priming procedure to be used in this task: The main methodological departure from lexical decision is that the referent is presented just above, and at the same time as the forward mask, and instead of deciding whether the target is a word or not, the decision is whether the target is the same or different from the referent. Thus, unlike the lexical decision task, the task does not require lexical retrieval (i.e., the decision requires whether the target matches the presented referent, not whether it matches an item(s) in the reader’s lexicon) and accordingly, priming in this task is insensitive to factors relevant to lexical retrieval such as the lexical status of targets and word frequency (Norris & Kinoshita, 2008) and the consonant–vowel status of the prime (Perea & Acha, 2009) (for detailed discussion of task comparison and simulation of priming based on the Bayesian Reader framework, see e.g., Norris & Kinoshita, 2008; Norris et al., 2010; also Kinoshita & Norris, 2012). Kinoshita and Norris (2009) showed that the masked priming effect in this task is insensitive to the visual similarity of the prime and target words presented in different case (e.g., edge and EDGE are visually dissimilar; kiss and KISS are visually similar), indicating that priming in this task is based on abstract letter representations, just as in the lexical decision task (as shown by Bowers, Vigliocco, and Haan (1998)). Kinoshita and Norris further demonstrated that the same-different task shows robust TL priming effects (see also e.g., García-Orza, Perea, & Muñoz, 2010; Perea & Acha, 2009, for replications) but also that priming was reduced greatly for a prime in which letter order was completely “scrambled” (e.g., *ifhat-FAITH*). This means that the task is sensitive to letter order. These features make the cross-case same-different task suitable for investigating orthographic processing.

There are several reasons for preferring the same-different task to the lexical decision task for the present purpose. One is that the same-different task typically yields a larger priming effect than the lexical decision task (see, e.g., Norris et al., 2010). This is expected from the fact that the task requires a decision about the match between the target and a single referent rather than a match between the target and representation(s) in the lexicon as required by the lexical decision task. The bigram primes are expected to yield small priming effects as indicated by the small match values computed by the OB models when the target word is long (which is necessary to test the assumption concerning the number of intervening letters in an OB). It is therefore important that the task is sensitive enough to pick up the small priming effects.

Second, it is now well-established that masked priming effects in lexical decision are sensitive to factors other than orthographic similarity (Guerrera & Forster, 2008; Kinoshita & Norris, 2009; Lupker & Davis, 2009). Orthographic priming effects in the lexical decision task are modulated by the lexical characteristics of the stimuli, in particular, by the neighborhood density of the target, with the priming effect being weak or absent for short words with many neighbors – which is referred to as the target density constraint (Forster, 1987). This is likely to limit the scope for observing orthographic priming effects with two-letter words as targets. In contrast, in the same-different task, orthographic priming is insensitive to neighborhood density (Kinoshita, Castles, & Davis, 2008), and it has been shown to be a more sensitive task for investigating small differences in orthographic similarity (e.g., Norris et al., 2010).

Third, the same-different task allows a more direct test of the OB model predictions. The main means of generating predictions from the OB models concerning priming is to compute “match scores”, which index the orthographic similarity between two letter strings based on the number of OBs shared by the prime and target.^3^ As noted, masked priming effects in lexical decision are sensitive to lexical variables, but the match scores are not. Given that masked priming effects in the same-different task are also insensitive to lexical variables like lexical status and neighborhood density, and the OB models have yet to implement the influence of such factors, this task is more suited to testing the predictions of the OB models based on match scores.

Fourth, unlike the lexical decision task, the same-different task can be used to test masked priming with a small set of targets repeatedly. This point was noted by Kinoshita and Kaplan (2008) who investigated masked priming of single letter stimuli. Earlier, Bowers et al. (1998) used the alphabet decision task and the vowel–consonant decision task to investigate priming of abstract letter identities. They compared the size of identity priming effect for prime–target pairs in different case which are either visually similar (e.g., c-C, x-X) or visually dissimilar (e.g., a-A, g-G). The priming effect was small, and was statistically non-significant for visually dissimilar letter pairs, forcing the authors (against other evidence acknowledged by the authors as suggesting to the contrary) to conclude that there were no abstract letter identities capable of supporting priming. In contrast, Kinoshita and Kaplan found robust identity priming effects equal in size for visually similar pairs and dissimilar pairs. They argued that in tasks like the alphabet decision and vowel–consonant decision, subjects can learn to associate the response to the stimulus, and this stimulus–response mapping process can dominate the priming effect when a small set of stimuli are used repeatedly (cf. Damian, 2001). In the same-different task, the same stimulus can be used in the Same and Different trials, thus precluding the mapping of a stimulus to a specific response. This feature of the same-different task is particularly important for Experiment 1 which used two-letter words as targets, as the number of two-letter words is limited and stimulus repetition cannot be avoided.

It should be noted that in the same-different task, only the Same trials show masked priming effects, and not the Different trials. Norris and Kinoshita (2008, see also Kinoshita & Norris, 2012) explained this within the Bayesian Reader theory of masked priming as follows. Consider a trial requiring the “Same” response, e.g., where the referent is “cat”, and the target is “CAT”. An orthographically related prime (e.g., “ct”) will contribute evidence supporting the decision that the target is the same as the referent. An unrelated prime (e.g., “ge”) will contribute to a “Different” decision. The net result is a priming effect when comparing an orthographically related prime vs. an unrelated prime. Now consider a “Different” trial (e.g., where the referent is “pun” and the target is “CAT”). A prime orthographically related to the *target* (e.g., “ct”) contributes to the decision that the target is different from the *referent*. However, an unrelated prime (e.g., “ge”) also contributes to the decision that the target is different from the referent. The net result is no difference between an orthographically related prime and an unrelated prime, i.e., no priming effect. It is worth noting that the same principle explains why priming is absent for nonword targets in the lexical decision task, and also that both the absence of priming for Different decisions in the same-different task and for nonword targets in the lexical decision task are not due to the operation of a bias to respond “No” counteracting the benefit contributed by a related prime (for a detailed explanation and empirical evidence, see e.g., Kinoshita & Norris, 2010, 2011; Norris & Kinoshita, 2008).

To recap, in the present study we evaluated the two core assumptions of the OB models by investigating masked priming using bigram primes in the same-different task. The assumption that the order of letters in a word is coded by the presence of ordered letter pairs was examined in Experiments 1 and 3 by testing whether priming is present for reversed bigram primes (e.g., fo-OF, sb-ABOLISH). The assumption that OBs can span only up to two intervening letters was tested in Experiments 2 and 3 by manipulating the number of intervening letters in a bigram primes (e.g., bo-ABOLISH, bs-ABOLISH).

## Experiment 1

**Fig. 1:**
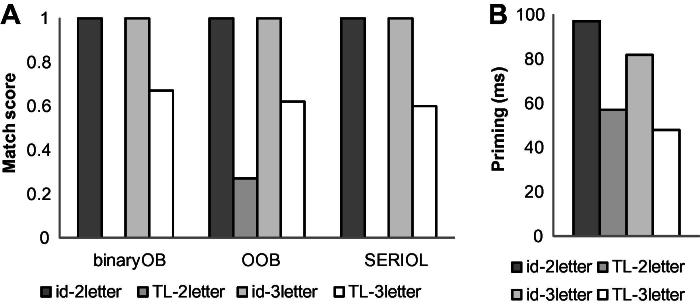
Match scores (a) and priming effects (b) for prime–target pairs used in Experiment 1.

In Experiment 1, our aim was to determine whether transposed-letter priming would be obtained with two-letter words. According to the open bigram models – with the exception of the OOB model which incorporates positional noise and hence predicts a small priming effect for contiguous reverse bigrams – there should be no TL priming effect, because two-letter words (e.g., OF, MY) do not share any OBs with two-letter TL primes (e.g., fo, ym). As a comparison condition and a manipulation check, we also included 3-letter words (e.g., THE primed by hte) which, according to all OB models, should show TL priming effects. Match scores computed from the binary OB model, the OOB model, and SERIOL are shown in Fig. 1a.

### Method

#### Participants

Twelve students from Macquarie University Psychology Research Participation Pool participated in Experiment 1 in return for course credit.

#### Design

Experiment 1 used the cross-case same-different matching task, and constituted a 2 (Word length: 2-letters vs. 3-letters) × 3 (Prime type: Identity vs. Transposed-letter, hereafter TL vs. all-letter-different, hereafter ALD) × 2 (Response: Same vs. Different) factorial design, with all factors manipulated within subjects. The dependent variables were response latency and error rate.

#### Materials

The critical stimuli were 20 two-letter words and 20 three-letter words with no repeated letters. As would be expected of short words, they were high-frequency words (626–69971, mean 8063 per million by Kucera & Francis, 1967, 12.15–16.96, mean 14.42 log HAL Frequency, and 723.8–41857.1, mean 7046.7 per million by Subtlex frequency, Brysbaert & New, 2009). The number of orthographic neighbours as defined by the “Coltheart’s N” metric (Coltheart, Davelaar, Jonasson, & Besner, 1977) ranged between 1 and 17 (mean 7.3).

For each word, three primes were generated. The *Identity* prime was the same word as the target, e.g., *of-OF, the-THE*. The TL prime had two adjacent letters transposed in position, e.g., *fo-OF*; for the three-letter words, this involved the first and the second letter, e.g., *hte-THE*. The ALD prime was the TL prime of another word so that there was minimal letter overlap, e.g., *ym-OF*, *nma-THE*. The critical target words and primes are listed in the Appendix.

Each target was presented six times, three times with the same referent word and three times with a different referent word (which was another target word of the same length), each paired with the three types of prime (Identity, TL, ALD). There was just one list version containing 240 trials. In addition, there were 24 practice and initial buffer trials, constructed in the same way as, but using different stimuli from the test stimuli. These items were not included in the analysis.

#### Apparatus and procedure

Participants were tested in groups of 1–4, seated approximately 40 cm in front of a CRT monitor, upon which stimuli were presented. Each participant completed 240 test trials consisting of 120 Same and 120 Different trials, presented in two half blocks with a self-paced break between the blocks, with a different random order generated for each participant.

Participants were instructed at the outset of the experiment that on each trial they would be presented with a word in lowercase letters followed by a word in uppercase letters, and their task was to decide whether the two words were the same, ignoring the difference in case, as fast and accurately as possible. They were instructed to press a key on a response pad marked “+” for *Same* and a key marked “−” for *Different* responses.

Stimulus presentation and data collection were achieved through the use of the DMDX display system developed by K.I. Forster and J.C. Forster at the University of Arizona (Forster & Forster, 2003). Stimulus display was synchronized to the screen refresh rate (13.3 ms).

Each trial started with the presentation of a referent word in lowercase letters, together with, and above a forward mask consisting of three # signs for 998 ms. The referent word disappeared, and the forward mask was replaced by the prime in lowercase letters presented for 53 ms. The prime was in turn replaced by the target presented in uppercase letters for a maximum of 2000 ms, or until the participant’s response. Participants were given a feedback (“Wrong response” message on the screen) only when they made an error on a trial.

### Results and discussion

In this and all subsequent experiments, RT was analyzed using the linear mixed effects model, treating subjects and items as crossed random factors. The analyses we report are based on RTs from correct trials requiring the SAME response (since, as noted above, “Different” trials are insensitive to masked priming). RTs shorter than 250 ms were excluded from analysis (in Experiment 1, 27 data points). The cutoff was determined by inspecting the Q–Q plots of inverse-transformed RT (1/RT), carried out to approximate a normal distribution. As a result of the cutoff procedure, there were 1320 data observations in Experiment 1. We multiplied 1/RT by −1000 to maintain the direction of effects (so that a larger invRT meant a slower response). We used lme4 (Bates, Maechler, & Dai, 2008) and languageR packages (Baayen, 2008) as described in Baayen (2008) implemented in R (R Development Core Team, 2008).

In the analysis of RT, we first tested a model including the Prime type and Target length and their interaction, Log HAL frequency, *N* (centered to avoid a spurious correlation between the intercept and slope – see Baayen, 2008), and previous trial RT as fixed factors, and Subject slopes (12) and Word intercepts (40) as crossed random factors: invRT ∼ Primetype * Target length + Log_HALfreq + *N* + prevRT + (Primetype|subj) + (1|word). *p*-Values were estimated using the Markov Chain Monte Carlo (MCMC) sampling method (with the default 10,000 samples) as implemented in the languageR package (Baayen, 2008). The model was progressively simplified by excluding each factor if it was non-significant and the more complex model did not fit the data better. In the initial model, the Primetype by Target length interaction (identity priming x target length: *t* = 0.093, *p* = .91; TL priming × target length: *t* = .833, *p* = 40), Target length (*t* = .125, *p* = .90), Log HAL frequency (*t* = .303, *p* = .73), *N* (*t* = −.565, *p* = .57) were all found to be non-significant, and as their inclusion did not improve the model fit to the data, the model we report included only the Primetype and prevRT as fixed factors and subject intercepts and word intercepts as crossed random factors: invRT ∼ Primetype + prevRT + (1|subj) + (1|word). Mean decision latencies and error rates are presented in Table 1; the priming effects relative to the ALD prime are shown in Fig. 1b.

In RT, both the identity priming effect (id < ALD, *t* = −16.276, *p* < .0001) and the TL priming effect (TL < ALD, *t* = −9.219, *p* < .0002) were highly significant. Critically, the TL priming effect for the 2-letter words (57 ms) was substantial, and there was no evidence that it was smaller than that for the 3-letter words (48 ms): As noted above, the interaction between TL priming and target length was non-significant. The identity prime condition was significantly faster than the TL prime condition, *t* = −7.19, *p* < .0002. The effect of previous trial RT (*t* = 5.205, *p* < 0.0001) was also highly significant.

Accuracy data (using the logistic regression model) were also tested using the same model, excluding the prevRT factor: Accuracy ∼ Primetype * Targlength + Log_HAL-freq + cOrthN + (1|subj) + (1|word). As the effects of Log HAL frequency (*z* = −.012, *p* = .99), and *N* (*z* = .675, *p* = .499) were non-significant, they were excluded, and the final model included Primetype and Targetlength and their interaction as fixed factors: Accuracy ∼ Primetype * Target length + (1|subj) + (1|word). In this model, the identity priming effect was significant, *z* = 3.102, *p* < .002, but not the TL priming effect, *z* = .669, *p* = .50. However, the latter was qualified by an interaction with Target length, *z* = 2.045, *p* < .05. The interaction reflected a greater TL priming effect for the 3-letter words (8.3%) than for the 2-letter words (1.7%). The TL priming effect for 3-letter words was significant, *z* = 3.274, *p* < .01, but not for 2-letter words, *z* = 0.664, *p* = .50.

The results were clear: Two-letter words like OF and MY showed robust TL priming effects, even though the TL prime and the target share no OBs. This is clearly at odds with all OB models, except the OOB model. The OOB model predicts a small priming effect for reversed contiguous bigrams because it incorporates positional noise. However, even the OOB model greatly underestimates the size of TL priming effect for the two-letter words, predicting it to be substantially smaller than for three-letter words (match score values are .27 for two-letter words and .62 for three-letter words), while the results showed no statistical difference between the two. Neither the Binary OB model (Grainger & van Heuven, 2003) nor the SERIOL model (Whitney, 2001, 2008) accommodates the finding of transposed-letter priming effect for two-letter words.

Note that these results cannot be explained by assuming that the priming effects here were due solely to the priming of letter identities. As noted earlier, in the same-different task priming is greatly reduced for primes sharing the same letters as the target but in a completely different order (Kinoshita & Norris, 2009), indicating that it is sensitive to letter order. Here too, the priming effect was substantially (and significantly) reduced for primes in which the letter order was distorted (the TL prime) than for the prime containing the letters in the canonical order (the identity prime).

It should be noted however that the finding with two-letter words may be limited in generalizability. For one thing, there are a limited number of two-letter words, and the fact that each item had to be used repeatedly with different primes is not ideal. Moreover, the transposition necessarily involved edge letters (first and final letter of a word) which are known to behave differently (although generally showing reduced, rather than enhanced, TL priming effects). In subsequent experiments we will therefore use longer (7-letter) words, manipulating only word-internal letters.

## Experiment 2

In Experiment 2, we test another core assumption of OB models, namely, that OBs cannot span more than two intervening letters. To this end, we used 7-letter words (e.g., ABOLISH) and bigram primes that spanned 0, 1 or 3 letters (0L, 1L, and 3L, respectively). All bigram primes consisted of word-internal letters (e.g., bo, bl, bs in ABOLISH).

**Fig. 2:**
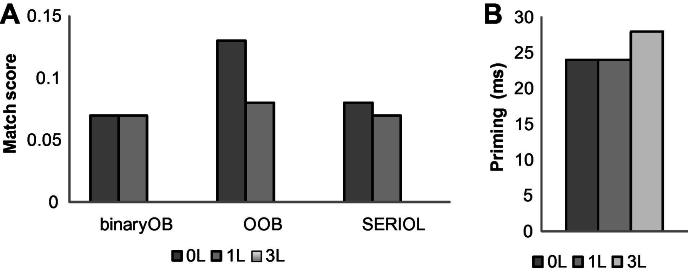
Match scores (a) and priming effects (b) for prime–target pairs used in Experiment 2.

As shown in Fig. 2a, all OB models predict no priming from 3L primes. They differ somewhat with regards the amount of priming produced by the 0L and 1L primes. The Binary OB model (Grainger & van Heuven, 2003) does not weight the distance between the letter pair and hence predicts equal priming with 0L and 1L primes. Both SERIOL and the OOB model weight contiguous OBs more and hence predict greater priming with 0L than 1L primes, however, the predicted difference is very small for SERIOL (match scores are .08 and .07 for 0L and 1L primes, respectively). For all models, the match scores are small, as a bigram prime matches just one out of 15 OBs (not counting the edge bigrams) in a 7-letter word.

### Method

#### Participants

An additional 32 students from Macquarie University Psychology Research Participation Pool participated in Experiment 2 in return for course credit.

#### Design

Experiment 2 had four prime conditions, with 3 OB conditions differing in the number of intervening letters (0, 1 or 3 letters), and the ALD control condition. The dependent variables were response latency and error rate.

#### Materials

The critical stimuli were 80 seven-letter words with no repeated letters, e.g., *ABOLISH, CHIMNEY*. They were low to medium frequency (2–49, mean 12.9 per million by Kucera and Francis (1967, 5.21–10.00), mean 7.83 log HAL Frequency, and .14–153.12, mean 7.56 per million by Subtlex frequency). The number of orthographic neighbors (*N*) ranged between 0 and 3 (mean 0.47).

For each word, four primes were generated. The 0L prime was the two internal, adjacent letters in positions 2 and 3 or positions 5 and 6, e.g., bo-ABOLISH, is-ABOLISH. The 2L prime was the two internal letters that spanned one intervening letter, in positions 2 and 4 or positions 4 and 6, e.g., bl-ABOLISH, ls-ABOLISH. The 3L prime was the two internal letters in positions 2 and 6, e.g., bs-ABOLISH. The ALD prime was two letters not contained in the target, e.g., we-ABOLISH. The critical target words and primes are listed in the Appendix.

Within a list, each target was presented twice, once with the same referent word (e.g., referent – abolish, target – ABOLISH) and once with another word of the same length that did not share the same letters as the target (e.g., referent – thickly, target – ABOLISH). The 80 target words were divided into four sets and the assignment of sets to the four prime conditions was counterbalanced so that within a list a target word was paired with one prime type, and across every four lists it was paired with all four prime types. Each participant was presented with 160 test trials (80 “same” and 80 “different” trials). In addition, there were 10 practice and initial buffer trials, constructed in the same way as, but using different stimuli from the test stimuli. These items were not included in the analysis.

#### Apparatus and procedure

They were identical to Experiment 1.

### Results

The analysis of RT and error rate followed the same procedure as for Experiment 1. As in Experiment 1, only the *Same* responses were analyzed. (The descriptive statistics for the Different responses are shown in Table 2.) The RT trimming procedure excluding RTs shorter than 250 ms affected 2 data points in Experiment 2. There were 2440 data observations in the analysis of correct RT in Experiment 2.

As in Experiment 1, we used linear mixed effect modelling with −1000/RT (invrt) as the dependent variable. The initial model included as predictor variables Prime type, and the lexical factors, Log_HAL-frequency and *N* (centered), and previous trial RT (prevRT) as fixed factors and Subject slopes (32) and Word intercepts (80) as crossed random factors (invRT ∼ Primetype + Log_HAL-freq + *N* + prevRT + (Primetype|subj) + (1|word). As the comparison with the simpler model that included Subject intercepts did not significant improve the data fit (*χ*^2^(9) = 2.15, *p* = .98), we report the simpler model. As in Experiment 1, *p*-values were estimated using the MCMC sampling method. Mean response latencies and error rates of Experiment 2 are presented in Table 2; the priming effects relative to the ALD prime are shown in Fig. 2b.

In the first analysis, we included all prime conditions and used the ALD prime condition as the referent condition. All OB prime conditions were significantly faster than the ALD prime condition: OB0L < ALD, *t* = −4.702, *p* < .001; OB1L < ALD, *t* = −5.617, *p* < .001; OB3L < ALD, *t* = −4.978, *p* < .001. Effects of Log HAL frequency (*t* = −1.817, *p* = .067) and *N* (*t* = 1.47, *p* = .146) were non-significant. The effect of previous trial RT was highly significant, *t* = 9.922, *p* < .0001. Comparison between the three OB prime conditions showed no difference among them: OB0L vs. OB1L, *t* = .91, *p* = .376; OB1L vs. OB3L, *t* = .626, *p* = .542.

Accuracy data (using the logistic regression model) showed no effect of Log HAL frequency, *N*, or any difference between the prime conditions.

The main finding of Experiment 2 is the robust priming effect for bigram primes spanning three letters in the target (e.g., bs-ABOLISH), which is at odds with all OB models. The results also showed that the number of intervening letters in an OB had no effect on the size of priming: Contiguous OBs and non-contiguous OBs spanning one or three intervening letters produced the same amount of priming. The absence of an effect of distance is inconsistent with all open bigram models except the Binary OB model. However, as noted earlier, Grainger and van Heuven (2003) regard this aspect of the model as “a simplification of what we expect to be a continuous decrease in bigram activation as a function of the distance separating the component letters” (p. 15) rather than an essential assumption. In any case, the complete absence of an effect of distance across 0–3 intervening letters is inconsistent with all OB models, including the Binary OB model.

## Experiment 3

Experiment 1 showed that reversing the order of letters in a bigram prime did not eliminate priming, and Experiment 2 showed that bigram primes spanning the distance of three letters produced robust priming. Experiment 3 combined the reversal and distance manipulations. As in Experiment 2, the targets were 7-letter words, and the bigram primes were all word-internal. The bigram primes were either in the canonical order or in reversed order (rev), and were either contiguous bigrams (0L) or non-contiguous bigrams spanning three letters (3L), resulting in four experimental prime conditions: (1) 0L (e.g., bo-ABOLISH), (2) 3L (e.g., bs-ABOLISH), (3) rev0L (e.g., ob-ABOLISH), (4) rev3L (e.g., sb-ABOLISH).

**Fig. 3:**
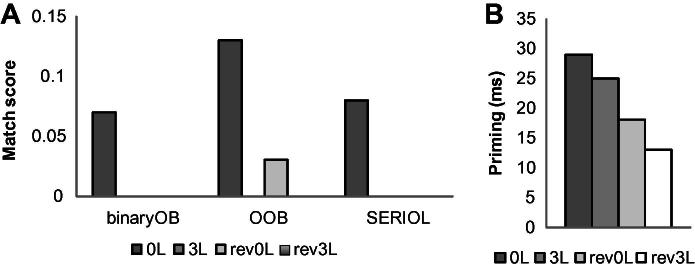
Match scores (a) and priming effects (b) for prime–target pairs used in Experiment 3.

As shown in Fig. 3a, both the Binary OB model and SERIOL predict priming only for the 0L prime. The OOB model in addition predicts a small priming effect for rev0L, smaller than that for 0L.

### Method

#### Participants

An additional 30 students from Macquarie University Psychology Research Participation Pool participated in Experiment 3 in return for course credit.

#### Design

Experiment 3 had five prime conditions, with the four experimental conditions resulting from a factorial combination of Distance (0L vs. 3L) and Order (canonical vs. reversed), and the ALD control condition. The dependent variables were response latency and error rate.

#### Materials

The critical stimuli were 100 seven-letter words with no repeated letters, e.g., *ABOLISH, CHIMNEY*. They were selected in the same way as the words used in Experiment 2, and were low to medium frequency (2–49, mean 12.0 per million by Kucera and Francis (1967, 5.21–10.00), mean 7.79 log HAL Frequency, and .14–153.12, mean 6.67 per million by Subtlex frequency). The number of orthographic neighbors (*N*) ranged between 0 and 3 (mean 0.46).

For each word, five primes were generated. The 0L prime was the two internal, adjacent letters in position 2 and position 3, or position 5 and position 6, e.g., bo-ABOLISH, is-ABOLISH. The 3L prime was the two internal letters that spanned three intervening letters, i.e., in position 2 and position 5, e.g., bs-ABOLISH. The rev0L prime was the same as the 0L prime but with the letters in reversed order, e.g., ob-ABOLISH, si-ABOLISH. The rev3L prime was the same as the 3L prime with the letters in the reverse order, e.g., sb-ABOLISH. The ALD prime was two letters not contained in the target, e.g., we-ABOLISH. The critical target words and primes are listed in the Appendix.

Within a list, each target was presented twice, once with the same referent word (e.g., referent – abolish, target – ABOLISH) and once with a different referent word (which was another word of the same length that did not share the same letters as the target e.g., referent – thickly, target – ABOLISH). The 100 target words were divided into five sets and the assignment of sets to the five prime conditions was counterbalanced so that within a list a target word was paired with one prime type, and across every five lists it was paired with all five prime types. Each participant was presented with 200 test trials (100 “same” and 100 “different” trials). In addition, there were 10 practice and initial buffer trials, constructed in the same way as, but using different stimuli from the test stimuli. These items were not included in the analysis.

#### Apparatus and procedure

They were identical to Experiment 1.

### Results and discussion

The analysis of RT and error rate followed the same procedure as for Experiment 1. As in Experiment 1, only the *Same* responses were analyzed. (The descriptive statistics for the Different responses are shown in Table 3.) The RT trimming procedure excluding RTs shorter than 250 ms affected 1 data point in Experiment 3. There were 2869 data observations in the analysis of correct RT in Experiment 3.

As in Experiment 1, we used linear mixed effect modelling with −1000/RT (invrt) as the dependent variable, and as predictor variables Prime type, and the lexical factors, Log_HAL-frequency and *N*, and previous trial RT (prevRT) as fixed factors. We first compared a model that included Subjects slopes (30) and Words (100) as crossed random factors (invRT ∼ Primetype + Log_HAL-freq + *N* + prevRT + (prime|subj) + (1|word), and a model that included Subjects intercepts (30) and Word intercepts (100) as crossed random factors (invRT ∼ Primetype + Log_HAL-freq + *N* + prevRT + (1|subj) + (1|word). As the more complex former model did not improve the data fit (*χ*^2^(14) = 6.21, *p* = .96), we report the latter, simpler model. As in Experiment 1, *p*-values were estimated using the MCMC sampling method. Mean response latencies and error rates of Experiment 3 are presented in Table 3; the priming effects relative to the ALD prime are shown in Fig. 3b.

We first included all prime conditions and used the ALD prime condition as the referent condition. All experimental conditions showed priming: 0L < ALD, *t* = −5.355, *p* < .0001; 3L < ALD, *t* = −4.031, *p* < .0002; rev0L < ALD, *t* = −2.844, *p* < .001; and rev3L < ALD, *t* = −2.367, *p* < .02. There was no effect of Log HAL frequency, *t* = −.785, *p* = .455, or *N*, *t* = .469, *p* = .651. The effect of previous trial RT was highly significant, *t* = 8.619, *p* < .0001.

We then tested a model excluding the ALD condition to test the cost of bigram reversal and letter distance in a factorial design. The model tested Distance (0L vs. 3L) and Order (canonical vs. reversed) and their interaction, and the lexical factors, Log_HAL-frequency and *N*, and previous trial RT (prevRT) as fixed factors and Subjects (30) and Words (100) as crossed random factors: invRT ∼ Distance * Order + Log_HAL-freq + *N* + prevRT + (1|subj) + (1|word), using data that excluded the ALD prime condition (2307 observations). As before, there was no effect of Log HAL frequency, *t* = −1.156, *p* = .247, or *N*, *t* = −.126, *p* = .899. The effect of previous trial RT was highly significant, *t* = 7.657, *p* < .0001.The effect of Distance was non-significant, *t* = −1.36, *p* = .17, but Order was significant, *t* = 2.493, *p* < .02, and the interaction was non-significant, *t* = −.628, *p* = .53. Thus, priming was sensitive to order, but not the distance (the number of letters) between the letters in the bigram prime, and irrespective of distance, order reversal reduced the amount of priming (by 12 ms).

In the analysis of accuracy, there were 3000 observations. We tested the model (Accuracy ∼ Primetype + (1|subj) + (1|word)), using the logistic model, including all prime conditions. Referent to the ALD condition, the 0L condition was more accurate, *Z* = 2.089, *p* < .04, as was the 3L condition, *Z* = 2.504, *p* < .02. Neither of the reversed condition differed from the ALD condition. We then analysed the prime conditions excluding the ALD conditions as a factorial design, testing the model (Accuracy ∼ Distance * Order + (1|subj) + (1|word)). In this model, neither Distance, Order or the interaction had significant effects.

To sum up, Experiment 3 used 7-letter words and tested the combined effects of letter distance and reversal, using word-internal bigrams. The results replicated the robust priming effects for reversed contiguous bigrams observed in Experiment 1, and for bigrams spanning three intervening letters observed in Experiment 2. This experiment also replicated the absence of distance effect observed in Experiment 2. All of these findings are inconsistent with all open bigram models. This experiment in addition showed priming for the rev3L prime, a bigram containing letters that span three intervening letters in reverse order. This rules out even the models (e.g., Dehaene et al.’s LCD model, 2005; Garinger et al.’s OOB model, 2006) that incorporate positional noise and hence predict a small priming effect from reverse primes provided that the letters are contiguous. Consistent with Experiment 1, priming was smaller for reversed primes, indicating that priming in this task was sensitive to letter order.

## General discussion

The present study evaluated two core assumptions of open bigram (OB) models in coding the order of letters in words. An OB is an ordered letter pair which may be contiguous or non-contiguous. OB models posit that letter order in a word is represented by a set of ordered letter pairs, for example, CAT is represented as {CA, CT, AT}. Accordingly, a key prediction of OB model is that there should be no priming for reversed OBs (e.g., TC in CAT). Contrary to this prediction, robust priming effects were observed with reversed bigram primes. The fact that priming was found for non-contiguous reversed bigrams spanning three letters (e.g., SB in ABOLISH) rules out even the models which incorporate positional noise (Dehaene et al., 2005; Grainger et al., 2006) and hence predict priming for reversed bigrams but only for contiguous letter pairs.

Another assumption shared by all current OB models is that the number of intervening letters in an OB is limited to two, e.g., the open bigram JE is not represented in JUDGE. Contrary to this assumption, robust priming effects were observed with bigram primes spanning three intervening letters (e.g., bs-ABOLISH). The results also showed no graded effects of distance: Contiguous OBs (e.g., bo-ABOLISH) and non-contiguous OBs spanning three intervening letters produced the same amount of priming. The absence of distance effect is at odds with all OB models: Even the Binary OB model regards the non-graded effect of distance as a “simplification” (Grainger & van Heuven, 2003, p. 15).

These results challenge the core assumptions of open bigram models. These assumptions are not parameter-dependent but are central to the notion of open bigrams and shared by all OB models; modification to these assumptions would amount to giving up the essence of open bigrams.^4^ Note also that the results cannot be dismissed by arguing that the predictions were based on match scores and that fully implemented OB models may make different predictions. The match scores are more than just an approximation to what a full model might produce. In the case of the manipulations we test, the match scores determine which orthographic representations are available to drive latter stages of processing and therefore which patterns could possibly produce priming using that particular OB representation as input. This is most apparent in Experiment 1. FO cannot possibly prime OF in a model where the orthographic input takes the form of OBs because the two have nothing in common. Given that input, nothing that might happen subsequently in a model could make FO and OF become similar – that information has been thrown away.

In contrast to the open bigram models, the presence of priming for the reverse bigram primes and bigram primes spanning three letters can be accommodated readily by both our noisy channel model (Norris & Kinoshita, 2012) and Davis’ (2010) Spatial Coding model. The noisy-channel model explains orthographic priming in terms of the evidence contributed by the prime that is consistent with the target sequence, based both on the letter identity and letter order information sampled from the perceptual input. A bigram prime comprised only of letters present in the target would obviously contribute more evidence than a prime comprised of letters not in the target. The spatial configuration of the letters in the input provides the information about letter order, and for any two letters presented in close spatial proximity, as in the bigram primes used in the present experiments, the order information is fairly ambiguous because of positional noise. Thus, reversing the order of letters in a bigram prime would result in only a small reduction in letter order information, as was observed to be the case. In the noisy channel model, uncertainty in the location of input letters means that for any pair of adjacent letters in the prime, there is some possibility that the perceptual evidence was actually generated by those letters in the reversed order. This effect operates at the level of the letters in the *prime*. This uncertainty in order emerges regardless of whether the corresponding letters in the *target* are adjacent, or far apart (i.e., whether the bigram prime was ‘bo’ in ABOLISH or ‘bs’ in ABOLISH). Accordingly, there should be little effect of the number of intervening letters on the size of priming, as was found to be the case.^5^

According to the Spatial Coding model, the amount of priming is determined by the degree of similarity of the spatial gradient representations of the prime and target. The spatial gradient represents the order of the letters present in the string, with the first letter having the highest activation, and each subsequent letter having progressively lower level of activation. The letters not present in the string has no activation in the spatial gradient representation, hence an ALD prime cannot have any similarity to the target; in comparison, the spatial gradient of a letter string containing two letters present in the target has some overlap with the spatial gradient of the target. Thus, the priming found with the critical primes (the reverse prime and the prime spanning three intervening letters) is a straightforward prediction of the Spatial Coding model. The transposition of two adjacent letters alters the spatial gradient representation only a little, thus the Spatial Coding model predicts some decrement but not a complete elimination of priming as a result of reversal, like the noisy channel model. For the distance manipulation, unlike the noisy channel model, the Spatial Coding model predicts more priming for the 0L prime than for the 3L prime (the match scores are .22 and .12, respectively). This is due to the fact that in the Spatial Coding model, orthographic similarity is determined by the physical similarity of the spatial configurations of the letters. The two letters in the bigram primes were always spatially adjacent. Accordingly, the spatial configuration of the prime (e.g., bo or bs) would be physically more similar to the spatial configuration of the same two letters that are also adjacent in the target (e.g., ABOLISH) than the letters that are further apart, spanning intervening letters in the target (e.g., ABOLISH). In other words, in the Spatial Coding model, similarity of the spatial configuration of the letters directly maps onto orthographic similarity, in line with what Davis (2006) referred to as “perceptual correspondence” (p. 183). These differences notwithstanding, both the noisy channel model and the Spatial Coding model predict non-zero priming for reversed bigram primes and bigram primes spanning three intervening letters in the target. This is because unlike the open bigram models, in these models the coding of letter order is not an all-or-none affair dependent on the presence of representations dedicated to coding the relative order of two letters that occur close together in a word– representations that code local order.

### Problems with local context coding

As just noted, open bigrams differ from the alternative models in using local context to code letter order. Grainger and van Heuven (2003; Grainger & Ziegler, 2011) acknowledged the works by Wickelgren (1969) and Mozer (1987) who also proposed local context-dependent representations as the inspiration for their OB proposal. In Wickelgren’s scheme, dubbed the Wickelcode, a letter is coded with respect to the immediately preceding and immediately succeeding letter (and a space #), i.e., a Wickelcode is an ordered letter triplet. Mozer (1987) extended the Wickelcode to include non-adjacent letters (e.g., NA_I where _ indicates any letter), like open bigrams. In all these schemes, a word is coded as an unordered set of these context-dependent representations, e.g., the word CAT is represented as a set of Wickelcodes {#CA, CAT, AT#}.

There have been previous attempts to use these context-dependent representations in connectionist models of visual word recognition (BLIRNET – Builds Location-independent Representations, Mozer, 1987) and reading aloud (Seidenberg & McClelland, 1989). It is noteworthy that these models were subsequently abandoned, due to problems associated with the nature of the input/output representations. It is therefore instructive to consider what these problems are.

One is that these representations lead to an explosion in the number of connections. Mozer (1987) noted that in BLIRNET, which used letter triplets that included non-contiguous letters, there were 56,966 possible representations. Even if not all such representations are required to code known words in a language (and also the number of required open bigrams would be fewer than the number of letter triplets), within a connectionist framework, the number of connections between the input and output units would increase enormously compared to when there are only 26 letter representations. This may perhaps explain why there has been no large-scale model of visual word recognition implementing open bigram representations.^6^

A related, well-known problem with Wickelcodes, pointed out by Plaut, McClelland, Seidenberg and Patterson (1996) in relation to the Seidenberg and McClelland’s (1989) model of spelling-to-sound mapping is the “dispersion problem”. Generalizing the spelling-sound mapping to novel instances (i.e., generating pronunciation for nonwords) was particularly poor in this model because the mappings (e.g., the sound associated with the letter P to the phoneme /p/) are dispersed over a large number of local contexts (e.g., _*PA*, *ELP*, *OP_*, etc.). It is of relevance that Goswami and Ziegler (2006) have noted the same problem with OB representations in learning to map orthographic representations to phonological representations. Grainger and Ziegler (2011) acknowledged this problem, and proposed a “dual orthographic code hypothesis”, ruling out OB representations in the sublexical assembly of phonology. Similarly, Whitney and Cornelissen (2008) noted that their OB representation is “taken to be specific to the lexical route” (p. 16), acknowledging that OBs are unsuited to sublexical generation of phonology.

There is another, more fundamental problem with Wickelcodes, pointed out by Pinker and Prince (1988). It is that two words of different length containing repeated Wickelcodes cannot be distinguished, because a whole word is represented as an unordered set of such representations, and hence repeated Wickelcodes are counted only once. The following example was given by Pinker and Prince: in Oykangand, an Australian language, *algal* means “straight”, and *algalgal* means “ramrod straight”, i.e., they are different (albeit semantically related) words, but contain the same Wickelcodes and hence cannot be distinguished. Exactly the same problem occurs with OBs: Letter strings that contain repeated OBs cannot be distinguished. For example, the Spanish word *CASA*, meaning house, contains the following OBs: {(#C), CA, CS, AS, AA, SA, (A#)}. This set is fully contained within the set of OBs (counting only OBs spanning up to 2 letters) representing the word *CASACA* meaning jacket: {(#C), CA, CS, AS, AA, SA, AC, (A#)}. Accordingly, in both SERIOL and the Binary OB model – the models that do not incorporate an additional positional noise assumption – the input *casaca* fully matches *CASA*: The prime–target pair *casaca-CASA* has a match score of 1.0, the same as that between *casa-CASA* and *casaca-CASACA*. This is not a unique example: The same problem is seen with the prime–target pairs *patata* (meaning potato) – *PATA* (paw), and *batata* (yam/sweet potato) – *BATA* (bathrobe/housecoat), just to name a few. As noted by Pinker and Prince, preserving distinctions that exist in a language would be an important criterion for assessing the adequacy of a representation, and Wickelcodes and open bigrams are clearly inadequate in this regard.

## Conclusion

Open bigram representations were proposed originally as a convenient computational solution to explain transposed-letter priming and relative-position priming effects (Grainger & Whitney, 2004), but there are now alternative models (the Spatial Coding Model, Davis, 2010; the noisy channel model, Norris & Kinoshita, 2012) that can readily simulate these effects without assuming open bigram representations. The noisy channel model has also provided large-scale simulations of lexical decision data of tens of thousands of words from the lexicon projects in English (the English Lexicon Project/ELP, Balota et al., 2007; the British Lexicon Project/BLP, Keuleers, Lacey, Rastle, & Brysbaert, 2012), French (Ferrand et al., 2010) and Dutch (Keuleers, Diependaele, & Brysbaert, 2010), unmatched by the open bigram models (see Norris & Kinoshita, 2012).

On open bigram representations, Dehaene (2009) wrote that “no one has ever seen bigram neurons” and “for the time being, they are a purely theoretical construction that cannot be tested directly” (p. 154). Grainger and Ziegler (2011) have recently acknowledged that “open-bigram coding is only one possible implementation of coarse-grained orthographic processing” (p. 4). The experiments reported here provide no evidence that letter order is coded by open bigrams. Taken together with known problems with local context coding, it is perhaps time to abandon open bigrams.

## Figures and Tables

**Table:** 

Target	Identity	TL	ALD
Two-letter words			
OF	of	fo	ym
IN	in	ni	fo
HE	he	eh	ni
AS	as	sa	eh
AT	at	ta	os
OR	or	ro	ta
WE	we	ew	ro
SO	so	os	ew
DO	do	od	sa
ME	me	em	od
GO	go	og	em
TO	to	ot	si
IS	is	si	ot
IT	it	ti	eb
BE	be	eb	pu
BY	by	yb	og
AN	an	na	yb
IF	if	fi	na
UP	up	pu	fi
MY	my	ym	ti

**Table:** 

Target	Identity	TL	ALD
Three-letter words			
THE	the	hte	nma
AND	and	nad	eth
WAS	was	aws	tno
FOR	for	ofr	swa
HIS	his	ihs	rfo
HAD	had	ahd	wno
NOT	not	ont	dha
BUT	but	ubt	dan
ONE	one	noe	tbu
YOU	you	oyu	rhe
HER	her	ehr	eon
SHE	she	hse	nca
WHO	who	hwo	yan
OUT	out	uot	wne
CAN	can	acn	tou
NEW	new	enw	uyo
TWO	two	wto	esh
ANY	any	nay	otw
NOW	now	onw	shi
MAN	man	amn	owh

**Table:** 

Target	0L	1L	3L	rev0L	rev3L	ALD
ABOLISH	bo	bl	bs	ob	sb	we
ANTIQUE	nt	ni	nu	tn	un	zo
CAPTIVE	ap	at	av	pa	va	um
CHIMNEY	hi	hm	he	ih	eh	ma
CRIMSON	ri	rm	ro	ir	or	sa
CRYSTAL	ry	rs	ra	yr	ar	ho
EDUCATE	du	dc	dt	ud	td	ml
EXHAUST	xh	xa	xs	hx	sx	ml
GHASTLY	ha	hs	hl	ah	lh	wd
GRIMACE	ri	rm	rc	ir	cr	tf
INDULGE	lg	ug	ng	gl	gn	mw
OBSCURE	ur	cr	br	ru	rb	ld
PLASTER	te	se	le	et	el	du
PROCURE	ur	cr	rr	ru	rr	dm
SCARLET	le	re	ce	el	ec	gu
SPARKLE	kl	rl	pl	lk	lp	mt
STEWARD	ar	wr	tr	ra	rt	lc
THUNDER	de	ne	he	ed	eh	fa
TRIUMPH	mp	up	rp	pm	pr	dc
URANIUM	iu	nu	ru	ui	ur	so
ACQUIRE	cq	cu	cr	qc	rc	mo
ANXIOUS	nx	ni	nu	xn	un	we
CHAMBER	ha	hm	he	ah	eh	vu
CLARIFY	la	lr	lf	al	fl	sd
CRUELTY	ru	re	rt	ur	tr	hs
DRASTIC	ra	rs	ri	ar	ir	me
EGOTISM	go	gt	gs	og	sg	qr
EXPLOIT	xp	xl	xi	px	ix	ju
GLAMOUR	la	lm	lu	al	ul	de
GRUMBLE	ru	rm	rl	ur	lr	sk
INHUMAN	ma	ua	na	am	an	ze
OLYMPIC	pi	mi	li	ip	il	da
PLUMBER	be	me	le	eb	el	da
PROFILE	il	fl	rl	li	lr	md
SCHOLAR	la	oa	ca	al	ac	mu
SPINACH	ac	nc	pc	ca	cp	vm
STOMACH	ac	mc	tc	ca	ct	dm
TRAGEDY	ed	gd	rd	de	dr	mf
TRUMPET	pe	me	re	ep	er	da
WHISPER	pe	se	he	ep	eh	fo
ADJUNCT	dj	du	dc	jd	cd	le
BLANKET	la	ln	le	al	el	dw
CHARIOT	ha	hr	ho	ah	oh	du
CLIMATE	li	lm	lt	il	tl	sd
CRUMBLE	ru	rm	rl	ur	lr	gy
DROUGHT	ro	ru	rh	or	hr	fx
ELASTIC	la	ls	li	al	il	wo
FORGIVE	or	og	ov	ro	vo	aq
GLIMPSE	li	lm	ls	il	sl	dm
IMAGERY	ma	mg	mr	am	rm	dw
INSPECT	ec	pc	nc	ce	cn	lz
OPTICAL	ca	ia	pa	ac	ap	ms
PREDICT	ic	dc	rc	ci	cr	fw
PROMISE	is	ms	rs	si	sr	dm
SHORTEN	te	re	he	et	eh	da
STADIUM	iu	du	tu	ui	ut	ro
STORAGE	ag	rg	tg	ga	gt	mv
TREASON	so	ao	ro	os	or	di
TWINKLE	kl	nl	wl	lk	lw	cs
WHISTLE	tl	sl	hl	lt	lh	br
AMONGST	mo	mn	ms	om	sm	ci
BLEMISH	le	lm	ls	el	sl	wv
CHARITY	ha	hr	ht	ah	th	xm
CLUSTER	lu	ls	le	ul	el	ma
CRUSADE	ru	rs	rd	ur	dr	mt
DYNAMIC	yn	ya	yi	ny	iy	wo
EMBARGO	mb	ma	mg	bm	gm	st
FRAGILE	ra	rg	rl	ar	lr	mt
GRAPHIC	ra	rp	ri	ar	ir	mt
IMPROVE	mp	mr	mv	pm	vm	cd
ISOLATE	at	lt	st	ta	ts	dm
ORGANIC	ni	ai	ri	in	ir	de
PREVAIL	ai	vi	ri	ia	ir	me
PRUDENT	en	dn	rn	ne	nr	wc
SLAVERY	er	vr	lr	re	rl	mo
STARLET	le	re	te	el	et	fo
THERAPY	ap	rp	hp	pa	ph	mv
TRICKLE	kl	cl	rl	lk	lr	md
UNLUCKY	ck	uk	nk	kc	kn	df
WRESTLE	tl	sl	rl	lt	lr	dn
ANGELIC	ng	ne	ni	gn	in	ko
BLISTER	li	ls	le	il	el	ga
CHERISH	he	hr	hs	eh	sh	wd
CRAVING	ra	rv	rn	ar	nr	fs
CRYPTIC	ry	rp	ri	yr	ir	sa
ECLIPSE	cl	ci	cs	lc	sc	md
ETHICAL	th	ti	ta	ht	at	qu
FRANTIC	ra	rn	ri	ar	ir	se
GRAVITY	ra	rv	rt	ar	tr	ms
IMPULSE	mp	mu	ms	pm	sm	dk
OBESITY	it	st	bt	ti	tb	km
PHANTOM	to	no	ho	ot	oh	ji
PRIVACY	ac	vc	rc	ca	cr	wd
PYRAMID	mi	ai	yi	im	iy	wo
SLUMBER	be	me	le	eb	el	fo
STAUNCH	nc	uc	tc	cn	ct	dm
THERMAL	ma	ra	ha	am	ah	fo
TRILOGY	og	lg	rg	go	gr	hm
UPRIGHT	gh	ih	ph	hg	hp	vc
WRINKLE	kl	nl	rl	lk	lr	sd

**Table 1:** Mean response latencies (RT, in ms) and percent error rates (%E) in Experiment 1.

	Target length					
	2-Letter target			3-Letter target		
Prime type	Example	RT	%E	Example	RT	%E
	Prime			Prime		
Same response	*of/OF*			*the/THE*		
Identity	*of*	380	2.1	*the*	389	4.2
Transposed letter	*fo*	420	7.5	*hte*	423	3.8
ALD	*ym*	477	9.2	*nma*	471	12.1
Identity priming effect		97	7.1		82	7.9
TL priming effect		57	1.7		48	8.3
Different response	*up/OF*			*was/THE*		
Identity	*of*	476	4.2	*the*	472	5.8
Transposed letter	*fo*	471	5.0	*hte*	475	2.9
ALD	*ym*	473	5.4	*mna*	501	3.3

**Table 2:** Mean response latencies (RT, in ms) and percent error rates (%E) in Experiment 2.

Prime type	Example	RT	%E		
	Prime			Priming effect	
Same response	*abolish/ABOLISH*				
0L	bo, is	463	4.2	24	0
1L	bl, ls	463	4.5	24	0
3L	bs	459	5.5	28	−1.3
ALD	du	487	4.2		

Different response	thickly/ABOLISH				
0L	bo, is	513	3.0		
1L	bl, ls	508	3.9		
3L	bs	515	3.6		
ALD	du	516	3.9		

**Table 3:** Mean response latencies (RT, in ms) and percent error rates (%E) in Experiment 3.

	Letter order in the bigram prime					
	Canonical			Reversed		
Prime type	Example	RT	%E	Example	RT	%E
	Prime			Prime		

Same response	*abolish/ABOLISH*					
0L	*bo, is*	455	3.7	*ob, si*	466	4.3
3L	*bs*	459	3.2	*sb*	471	4.2
ALD	*du*	484	6.3			

Priming effect						
0L		29	2.6		18	2.0
3L		25	3.1		13	2.1

Different response	thickly/ABOLISH					
0L	*bo, is*	498	2.5	*ob, si*	500	1.8
3L	*bs*	509	2.2	*sb*	501	1.8
ALD	*du*	500	2.2			

## A. Appendix

Critical stimuli used.

### A.2 Stimuli used in Experiment 2 and 3

Only the first 80 words were used in Experiment 2. Experiment 2 used the primes 0L, 1L and 3L; Experiment 3 used the primes 0L, 3L, rev0L and rev3L.

## Footnotes

1 It should be noted that whether the activation gradient is descending or ascending is arbitrary and has no theoretical bearing (see Davis, 2010, p. 716).

2 In addition to these models, Adelman (2011) has proposed the Letters in Time and Retinotopic Space (LTRS) model which also eschews slot-coding and can account for TL priming and relative position priming effects. We will not discuss this model further however, as its assumption regarding the issue investigated in the present study – whether letters are the only level of orthographic representation – is unclear (see p. 573, “What is the representation in LTRS”).

3 As in previous studies, we used the Matchcalculator available at Colin Davis’ website to compute the match values We thank Colin Davis for making the Matchcalculator available publicly. We note that Dehaene et al.’s (2005) LCD model does not specify parameter values and hence match score calculation is not available. However, this model is similar to the OOB model in its assumptions, and for present purposes the two may be considered equivalent.

4 One argument that might be used to defend OB representations is to claim that completely different orthographic representations are used in the same-different task and lexical decision task, and that the OB representations are used only in coding lexical representations. Not only is this unparsimonious (and limiting the explanatory power of OB models) but also the fact that robust TL priming effects are found for nonwords (Kinoshita & Norris, 2009; Perea & Acha, 2009) argues against this view.

5 In an apparent contradiction to the present finding, Perea, Dunabeitia, and Carreiras (2008) reported that in a lexical decision task priming was reduced for “distant” transposition where two letters intervened between the transposed letters (e.g., choaclcte-CHOCOLATE) relative to transposition of adjacent letters (e.g., chocloate-CHOCOLATE). This discrepancy is explained by the fact that in a TL prime containing all of the letters in the target, the order of letters in the whole string involving letters other than the transposed letters is changed, and that the change is necessarily greater when the transposition spans more intervening letters (e.g., in a string 1234567, the transposition of adjacent pairs 23 (1324567) does not affect the relative order of any other letters, but the transposition of 26 (1634527) changes the order of 2–3, 2–4, 2–5, 3–6, 4–6, 5–6 as well). In contrast, in a bigram prime (e.g., 23 or 26), there are only two possible orders (canonical – 2–3/2–6 – and reversed/transposed – 3–2/6–2).

6 Whitney (2012) has reported a simulation of words from the ELP database using a version of SERIOL. She used a subset (4008 4- to 6-letter words) selected from 6523 monomorphemic words of length 3- to 8-letters with log HAL frequency >4 and lexical decision accuracy >70%. This is a substantially smaller set than the total set (over 35,000 words in the ELP and 28,730 words in the British Lexicon Project) simulated by Norris and Kinoshita (2012) using the noisy channel model.
